# Cyclin K goes with Cdk12 and Cdk13

**DOI:** 10.1186/1747-1028-7-12

**Published:** 2012-04-18

**Authors:** Jiri Kohoutek, Dalibor Blazek

**Affiliations:** 1 Department of Toxicology, Pharmacology and Immunotherapy, Veterinary Research Institute, Hudcova 70, 62100 Brno, Czech Republic; 2 Central European Institute of Technology (CEITEC), Masaryk University, 62500 Brno, Czech Republic

**Keywords:** Transcription, Posttranscriptional processing, DNA damage, P-TEFb, Cyclin L, CTD code, CTD kinase, Phosphorylation of serine 2, BRCA1, ATR, FANCI, FANCD2

## Abstract

The cyclin-dependent kinases (Cdks) regulate many cellular processes, including the cell cycle, neuronal development, transcription, and posttranscriptional processing. To perform their functions, Cdks bind to specific cyclin subunits to form a functional and active cyclin/Cdk complex. This review is focused on Cyclin K, which was originally considered an alternative subunit of Cdk9, and on its newly identified partners, Cdk12 and Cdk13. We briefly summarize research devoted to each of these proteins. We also discuss the proteins' functions in the regulation of gene expression via the phosphorylation of serine 2 in the C-terminal domain of RNA polymerase II, contributions to the maintenance of genome stability, and roles in the onset of human disease and embryo development.

## Introduction

The family of cyclin-dependent kinases (Cdks) consists of 21 proteins whose activities usually require association with a specific cyclin subunit [1]. The first Cdks to be described were regulators of the cell cycle, such as Cdk1, Cdk2, Cdk4, and Cdk6. Their corresponding cyclins are also the most well characterized [2,3]. Another group of cyclin/Cdk complexes, including cyclin H/Cdk7 and cyclin T/Cdk9, have cell cycle-independent activities. These complexes are engaged in the regulation of transcription and posttrancriptional mRNA processing via the phosphorylation of the C-terminal domain (CTD) of RNA polymerase II (RNAPII) and other transcriptional regulators, such as DRB (5,6-dichloro-1-ß-D-ribofuranosylbenzimidazole) sensitivity inducing factor (DSIF) or negative elongation factor (NELF) [4]. Recent work led to the characterization of new transcription cycle-related Cdk complexes: cyclin K/cyclin-dependent kinase 12 (CycK/Cdk12) and CycK/Cdk13 [5,6]. In addition, it has been shown that CycK/Cdk12 maintains genome stability by regulating the expression of several important DNA damage response (DDR) genes [5,7]. These findings were fueled by recent developments in the field of RNAPII-mediated transcription that led to: 1) increased interest in the elucidation of the CTD code [4,8]; 2) the finding that promoter-paused RNAPII and elongation represent important regulatory steps in gene expression [9,10]; 3) the conclusion that phosphorylation of the CTD couples transcription to other cellular processes [11-13]; and 4) clarification of the relationship between what was considered to be the only human serine 2 (Ser2) CTD kinase, Cdk9, and its two putative yeast homologs, Bur1 and Ctk1 [6,14].

## A brief history of CycK, Cdk12, and Cdk13

### CycK

Human CycK was first identified as a protein that can rescue the lethality caused by deletion of the G1 cyclin genes *CLN1*, *CLN2*, and *CLN3 *in *Saccharomyces cerevisiae *[15]. It was discovered as a 40-kDa and 357-amino acid protein whose mRNA is ubiquitously expressed in all tested human and mouse tissues, and most abundantly in testis and ovaries [15]. Although at the time its relevant Cdk was not known, its association with RNAPII and potent *in vitro *and *in vivo *kinase activity on the CTD of RNAPII was well documented [15]. This activity was later associated with Cdk9, which was identified as a CycK interacting partner in a yeast two-hybrid assay [16]. Since then, CycK has long been considered to be an alternative cyclin subunit of Cdk9, together with CyclinT1 (CycT1) and two forms of CyclinT2, CycT2a and CycT2b [17,18]. At that time, it was also well-established that Cdk9 [in complex with cyclin subunits, also called positive transcription elongation factor b (P-TEFb)], is a crucial regulator of transcriptional elongation via phosphorylation of Ser2 in the CTD of RNAPII [19,20]. The lack of interest in further characterizing the CycK/Cdk9 complex probably stemmed from the discovery that CycT1/Cdk9 is the only Cdk9 complex able to bind HIV Tat protein and support HIV transcription [21]. This finding led the large majority of Cdk9 research to focus on the CycT1/Cdk9 complex, while CycK (and also CycT2) was only marginally studied. The only major functional difference between CycK and the CycT1/T2 subunits was noted by the Peterlin lab: when these cyclins are artificially tethered to a promoter, CycK activated transcription only via RNA recruitment, while CycT1 and CycT2 by both, RNA and DNA recruitment [22]. The first hints that CycK might not be associated with Cdk9 came from several mass spectrometry studies that failed to identify CycK associated with human Cdk9 complexes [23,24]. This was followed by the discovery that *Drosophila *Cdk12 interacts with CycK and the notion that metazoan CycK protein sequences are most similar to Ctk2, a cyclin partner of Ctk1 kinase, a yeast ortholog of Cdk12 [6]. Finally, a recent study revealed that human CycK is a 70-kDa and 580-amino acid protein with a C-terminal proline-rich region [5]. It associates with Cdk12 and Cdk13 in two separate complexes, but not with its previously identified partner, Cdk9 [5].

### Cdk12 and Cdk13

Cdk12 and Cdk13 were identified in cDNA screens for cell cycle regulators. Because their cyclin partners were not yet known, they were initially named CRKRS [25] and CDC2L5 [26], respectively. They were found to be 1490- and 1512-amino acid proteins, respectively, with a conserved central CTD kinase domain and degenerate RS domains identified in their N- and C-terminal regions [25-27]. Cdk12 was shown to phosphorylate CTD of RNAPII, *in vitro *[25]. Based on the interaction of Cdk12 and Cdk13 with overexpressed Cyclin L (CycL), CycL was reported to be their regulatory subunit, and the same studies suggested a role in the regulation of alternative splicing [28,29]. However, recent studies have reported that the endogenous *Drosophila *Cdk12 and human Cdk12 and Cdk13 do not associate with CycL, but rather with CycK [5,6]. In humans (and likely in other higher organisms), CycK binds Cdk12 and Cdk13 in two separate complexes [5], while in *Drosophila*, the related paralog of Cdk13 is missing and there is only a CycK/Cdk12 complex [6,30,31].

*Drosophila *and human Cdk12 phosphorylate Ser2 in the CTD of RNAPII, *in vitro *and *in vivo *[5,6], and Cdk13 phosphorylates the CTD of RNAPII, *in vitro *[6]. The functional link between CycK and Cdk12 is strongly supported by the overlapping set of genes affected by the absence of CycK or Cdk12, and their common phenotypes leading to genomic instability [5,7]. The exact function of the CycK/Cdk13 is not known.

A study by Bartkowiak et al. also showed that yeast Ctk2/Ctk1 are homologs of CycK/Cdk12 (and Cdk13 in mammals) and that yeast Bur2/Bur1 are homologs of CycT/Cdk9 [6]. Since it was assumed for many years that Cdk9 is a major Ser2 kinase in metazoan cells [20] and that its Ser2 kinase activity is split in yeast between its two homologs, Ctk1 and Bur1 [14], these findings represent an important milestone in our knowledge of Ser2 kinases and their relevant cyclin subunits. Table 1 provides a summary of information on the transcription cycle-related Cdks, their cyclin partners, yeast homologs, and kinase activity on the CTD of RNAPII. Of note, we could not identify the previously described 40-kDa form of CycK [15] at the level of mRNA or protein and were unable to confirm any association of the 40- or 70-kDa forms of CycK with Cdk9 in several cell lines [5]. Although we cannot completely exclude the possibility that CycK interacts with Cdk9 at certain developmental stages or under certain physiological conditions, the conclusions of several publications that consider CycK a bona fide partner of Cdk9 should be evaluated cautiously.

**Table 1:** Transcription-cycle related Cdks and their cyclin partners, yeast homologs, and kinase substrates

Cdk	Other nomenclature	Yeast homolog	Cyclin	Kinase activity on the CTD of RNAPII
Cdk7	CAK	Kin28	CycH [32]	Ser5 [33,34]
				
	CAK1			Ser7 [34,35]
				
	STK1			
				
	MO15			

Cdk8		Srb10	CycC [36]	CTD [37]

Cdk9-42 kDa	PITALRE	Bur1 [6]	CycT1 [17,18] CycT2a/b [17,18]	Ser2 [20]

Cdk9-55 kDa			CycT1 [38]	

Cdk11-46 kDa			CycL1 [39]	
				
			CycL2 [39]	

Cdk11-58 kDa			CycL1 [39]	
				
			CycL2 [39]	
				
			CycD3 [40]	

Cdk11-110 kDa	PITSLRE	Ste20	CycL1 [39,41]	CTD [41]
				
	CDC2L2		CycL2 [39,41]	

Cdk12	CRKRS CRKS	Ctk1 [6]	CycK [5,6]	Ser2 [5,6]
				
	CRK7			
				
	PITAIRE			

Cdk13	CDC2L5	Ctk1 [6]	CycK [5]	CTD [6]
				
	PITAIRE			

## Domain composition of CycK, Cdk12, and Cdk13

**Figure 1:**
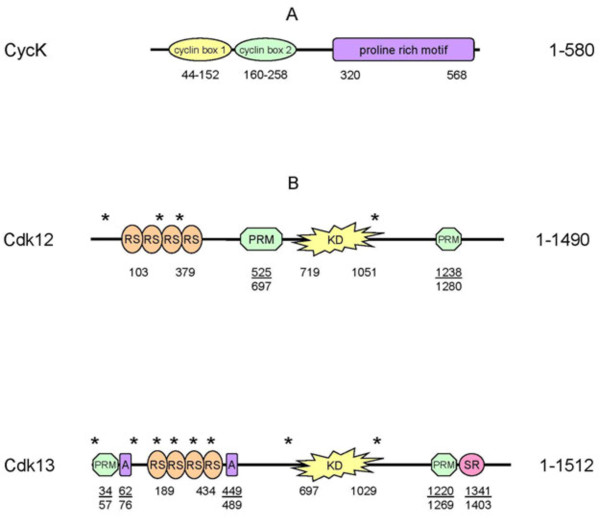
**Domain composition of CycK, Cdk12, and Cdk13**. A) A schematic representation of the CycK domain structure. Two cyclin boxes are depicted with a yellow and green ellipse, and the proline-rich domain by a violet oval. B) Schematic diagrams of Cdk12 and Cdk13 domain structures. Putative or verified nuclear localization signals (NLS) are depicted by asterisks. Arginine/serine-rich (RS), proline-rich (PRM), alanine-rich (A), and serine-rich (SR) domains are indicated by orange, green, violet, and purple ovals, respectively. A yellow asterisk represents the kinase domain (KD). Numbers below the schemes indicate the amino acid position for a given domain.

CycK has two N-terminal cyclin boxes and a C-terminal proline-rich region (Figure 1A). The N-terminal structure of CycK resembles the classical cyclin composition, with two cyclin boxes consisting of fifteen helices that mediate binding to a Cdk partner [42]. The newly described proline-rich region [5] consists of several proline-rich motifs (PRMs; Figure 1A). Proteins with PRMs are recognized for their function in transcriptional regulation, RNA processing, and alternative splicing [43].

The domain composition of Cdk12 is comparable to Cdk13 (Figure 1B). In both proteins, CTD kinase domain is localized in the center (Figure 1B), consists of about 300 amino acids, and their sequences are highly similar (> 93%). They contain a PITAIRE motif at the conserved position of the PSTAIRE motif found in yeast cdc2 and related kinases [25,26]. Like the cdc2 ATP-binding region, Cdk12 and Cdk13 also have characteristic threonine and tyrosine residues at the beginning of the ATP-binding region, implicating these residues in the regulation of the kinase activity. Both kinases also have a threonine in the activation 'T-loop' that is typically phosphorylated by a Cdk-activating kinase [25] and reviewed in [44,45].

There are 20 and 17 arginine/serine rich (RS) motifs in the N-terminus of Cdk12 and Cdk13, respectively (Figure 1B). RS domains serve as docking sites for the assembly of protein complexes and are found in splicing factors and regulators of splicing [46,47]. Cdk12, Cdk13, and CycK are localized in nuclear speckles, subnuclear structures enriched in mRNA splicing factors [5,25,28]. The common presence of the RS and CTD kinase domains in these Cdks makes them ideal candidates for coupling CTD phosphorylation with transcription and splicing [48-50]. Modulation of the level of Cdk12 and Cdk13 protein in cells affects the alternative splicing of certain splicing reporter constructs [27-29], and Cdk13 is suggested to be involved in the phosphorylation of ASF/SF2 and in the alternative splicing of HIV [51]. However, these studies involved the overexpression of Cdk12 and Cdk13, without their relevant cyclin partner, and thus, their direct roles in alternative splicing is still a matter of future research. Notably, using splicing-sensitive microarrays, we did not observe any significant splicing defects in several genes that are differentially expressed upon CycK or Cdk12 depletion [5].

Similar to CycK, PRMs are also present in the C-terminal region of both Cdks. In addition, Cdk12 carries one more PRM motif in its central region and Cdk13 has one in its N-terminus (Figure 1B). These PRMs may serve as binding sites for SH3, WW, or profilin domain containing proteins (reviewed in [52]). In contrast to Cdk12, the N-terminus of Cdk13 contains an alanine-rich motif with an unknown function. In addition, several putative or verified bipartite and non-bipartite nuclear localization signals have been described for both kinases [25,28].

## The CycK/Cdk12 complex phosphorylates Ser2 in the CTD of RNAPII

RNAPII directs the transcription of protein coding genes. The transcription process consists of several stages, including preinitiation complex formation, promoter clearance, pausing, productive elongation, and termination [53,54]. This transcription cycle is tightly linked to the co-transcriptional maturation of nascent transcripts, including pre-mRNA splicing and polyadenylation [13,55]. RNAPII contains an unstructured CTD with repeats of the evolutionarily conserved heptapeptide, Y_1_S_2_P_3_T_4_S_5_P_6_S_7_, where individual serines (Ser2, 5, and 7), threonine, and tyrosine can be phosphorylated [20,56-59]. Several Cdks and phosphatases regulate the phosphorylation status of the CTD and subsequent binding of transcription and pre-mRNA processing factors [4]. Patterns of phosphorylation (and other posttranslational modifications) of the CTD form the so-called "CTD code", which defines the action of RNAPII during the transcription cycle and directs the posttranscriptional processing of nascent transcripts [8]. Our knowledge of phosphorylation events on the CTD is based mostly on data obtained with phospho-specific antibodies. However, the reactivities of the antibodies are often affected by modifications on neighboring residues and the concentration used. In addition, they do not distinguish modifications among individual repeats [57,60,61] (*Saccharomyces cerevisiae *has 26, *Drosophila *has 44, and humans have 52 heptapeptide repeats [62]). Although these caveats should be taken into account (discussed below), some aspects of the CTD code and its role in the transcription cycle are relatively well established. Unphosphorylated RNAPII is recruited to the promoter for preinitiation complex assembly. Phosphorylation of Ser5 is a hallmark of paused RNAPII and is mediated by Cdk7 during initiation and promoter clearance. To release the paused RNAPII and allow productive elongation, Ser2 is phosphorylated by Cdk9. In early elongation, Ser5 residues are dephosphorylated, and the phosphorylation of Ser2 steadily accumulates to saturation while elongating on the transcription unit. Termination results in dephosphorylation of the CTD, which makes the RNAPII ready for another round of re-initiation (reviewed in [4,20,56,63]). How do the CycK/Cdk12 and CycK/Cdk13 affect phosphorylation of the CTD and regulate gene expression? For a long time, Cdk9 was considered to be the major elongation-associated Ser2 kinase in mammalian cells [20]. However, recent studies have found that Cdk12 phosphorylates Ser2, *in vitro*, and depletion of Cdk12 results in at least a fifty percent decrease in Ser2 levels in human cells [5,6]. Consistently, the requirement of Cdk12 for the bulk of Ser2 phosphorylation has also been documented in *Drosophila*, where Cdk12 also localized on several active genes, *in vivo*, predominantly in the middle and at the end of the transcriptional units [6]. In contrast, knockdown of Cdk13 did not produce any observable change in the levels of phosphorylated Ser2 [5,6]; however, subtle changes in the CTD phosphorylation were observed [6]. Although Ser2 phosphorylation is thought to be an important marker for the elongation of transcripts of most protein-coding genes [64-66], depletion of CycK and Cdk12 results in the downregulation of only a small subset of genes (predominantly long and complex ones) and in no change in the rate of global transcription [5]. However, the downregulated genes, including *breast cancer type 1 (BRCA1)*, *Fanconi anemia complementation group I *(*FANCI)*, and *ataxia telangiectasia and rad3-related (ATR) *had less RNAPII on their promoters and reduced amounts of nascent transcripts, which is indicative of a transcriptional defect [5]. Whether the diminished expression of a subset of genes is due to aberrant co-transcriptional processing, as suggested by the length and complexity of CycK/Cdk12-dependent genes, is not currently known. Notably, no global polyadenylation defects or splicing defects in most of the down-regulated genes were detected in the absence of CycK/Cdk12 [5].

A recent finding that Cdk12/13 and Cdk9 are homologs of yeast Ctk1 and Bur1, respectively, provided further insight into the possible role of Cdk12/13 and Cdk9 kinases in metazoans [6]. In yeast, Ctk1 is responsible for most of the Ser2 phosphorylation in promoter-distal regions and most of the Ser2 phosphorylation in bulk [67-69], while Bur1 contributes to the phospho-Ser2 marks at the 5' end of genes and the residual Ser2 phosphorylation [68,69]. Conspicuously, yeast deficient in Ctk1 or phosphorylated Ser2 do not have transcriptional defects [67,70,71], a finding consistent with results in mammalian cells depleted of CycK and Cdk12 [5]. These findings are surprising considering the well-recognized role of phosphorylated Ser2 in the regulation of transcriptional elongation [64-66]. However, there are alternative explanations. For example, upon depletion of Ctk1 or Cdk12, phospho-Ser2-specific antibody does not recognize phosphorylated Ser2 due to modification(s) of neighboring residues in the CTD repeats. In a different scenario, the absence of Ctk1 or Cdk12 results in different patterns of CTD phosphorylation, compatible with productive elongation. Alternatively, the functional outcome of phosphorylated Ser2 depends on which individual CTD repeat it is positioned on and by what kinase; phospho-Ser2 marks at certain CTD repeats deposited by the P-TEFb would direct transcription, while the ones at different CTD repeats deposited by the CycK/Cdk12 would be irrelevant for the efficiency of transcription of most genes.

It was also shown that Ser2 is phosphorylated during elongation by Ctk1, but Ctk1 is not required for association of elongation factors with transcribing RNAPII [70]. A study by Kim et al. shows that depletion of Ctk1 leads to the accumulation of RNAPII at the poly(A) sites of genes with good consensus poly(A) sites [71], while the distribution of RNAPII on other genes is unaffected [70,71]. This finding corresponds to the suggested role of Ser2 phosphorylation in 3' end RNA processing [70].

More insight into the function of phosphorylated Ser2, Cdk12, and Cdk9 was provided through the use of two phospho-Ser2-specific antibodies, H5 and 3E10. Whereas H5 predominantly recognizes phosphorylated Ser2 in the context of the phosphorylated neighboring Ser5 mark, 3E10 is more specific to CTD phosphorylated solely at Ser2 [60]. Loss of CycK/Cdk12 diminished the bulk levels of phosphorylated Ser2 to a similar extent when measured by both antibodies. The result was distinct from what was seen with depletion of Cdk9, where a smaller decline in Ser2 phosphorylation was observed when measured by the H5 antibody compared to the 3E10 antibody [5]. Interestingly, experiments in yeast suggest that there are two forms of Ser2 marks, one recognized by H5, which is dephosphorylated prior to termination, and another recognized by 3E10, which is dephosphorylated just after termination [72].

Because Ser2 phosphorylation is a marker of elongating RNAPII and is thought to be crucial for coupling transcription with mRNA-processing and other cellular processes, future studies untangling the physiological role of Cdk12 in these mechanisms promises to bring exciting findings.

## CycK/Cdk12 in the maintenance of genome stability

Genome stability is crucial for the viability of the cell and prevention of diseases, such as cancer, and is mediated by the DDR pathways [73,74]. Genome stability is maintained through the cooperation of hundreds of DDR proteins that detect lesions and mediate their repair [75,76]. Reparation of each type of DNA lesion requires the action of a specific group of DDR proteins. BRCA1, ATR, ataxia telangiectasia mutated (ATM), and Fanconi anemia proteins are at the core of several DDR pathways and are crucial for the maintenance of genome stability [77-79]. Many new players and cellular processes essential for the maintenance of genome stability have been identified from recent genome-wide screens [80]. Pathways and factors with little explored connection to DDR, including those involved in transcription and mRNA processing, were identified in several screens [75,76,81]. Notably, transcriptional cyclin-dependent kinases and phosphorylation of the CTD of RNAPII were functionally linked to the DDR and the maintenance of genome stability via regulation of transcription and mRNA processing [82,83].

Our work showed that the expression of several DDR genes, including some core players involved in the maintenance of genome stability, is CycK/Cdk12-dependent [5,7]. At least in the case of BRCA1, ATR, and FANCI, the regulation is at the transcriptional level [5,7]. In accordance with the observed down-regulation of many DDR genes, cells without CycK/Cdk12 induce spontaneous DNA damage signaling, as indicated by the accumulation of 53BP1 and γ-H2AX foci and an increased number of cells in the G2-M phase [5]. Cells depleted of CycK/Cdk12 are sensitive to various DNA damaging agents, including camptothecin, mitomycin C, and etoposide. These compounds cause various types of DNA lesions, and this sensitivity of CycK/Cdk12-depleted cells to various types of DNA damage is consistent with the proposed broad role of this complex in the DDR and maintenance of genome stability [5,7]. CycK was also independently identified in a genome-wide screen for proteins mediating resistance to the DNA damage-inducing compound, camptothecin [81].

A recent study by Yu et al. suggests a direct role for CycK in replication stress response [84]. Cells depleted of CycK show impaired cell cycle recovery after challenge with hydroxyurea and amphidicolin [84]. However, this result can also be explained by the indirect effect of decreased expression of ATR, the replication stress response regulator, in CycK-depleted cells [5]. The conclusion of this study is also complicated by the fact that CycK was studied as a cyclin subunit of Cdk9 [84]. Although a weak interaction of CycK with ATR was detected [84], the possibility of a direct role for CycK/Cdk12 in the replication stress response requires more research. Another line of evidence supporting a role for CycK in the DDR comes from the p53-dependent expression of CycK in response to treatment with adriamycin, ultraviolet, or gamma irradiation [85].

Supported by biochemical, functional, and the evolutionary characterization of the CycK/Cdk12 and CycT/Cdk9 complexes [5,6], it is conceivable that both contribute to the maintenance of genome stability, but through different mechanisms. The CycK/Cdk12 complex maintains genome stability through the regulation of DDR gene expression [5,7]. Consistently, its yeast homolog, Ctk2/Ctk1, is also implicated in the expression of several DDR genes, and a mutation in the Ctk1 kinase domain renders cells sensitive to DNA damage [83]. In contrast, the function of Cdk9 and its yeast homolog, Bur1, in the maintenance of genome integrity appears to be direct and independent of the modulation of DDR gene expression, as judged by results from genome-wide expression arrays [84,86]. Cdk9 was found in complex with replication stress response proteins ATR, ataxia telangiectasia and Rad3-related interacting protein (ATRIP), and claspin, and, upon replication stress, it localizes to chromatin to eliminate the collapse of stalled replication forks [84]. The kinase activity of Cdk9 seems to be essential for cell cycle recovery after replication stress, but whether the CTD of RNAPII or other Cdk9-associated proteins are substrates mediating this function is unknown [84]. Notably, the 55-kDa, but not the 42-kDa isoform, of Cdk9 was shown to associate with Ku70, a protein directly involved in DNA repair by non-homologous end-joining [87]. In yeast, Bur1 binds the Rfa1 protein that protects ssDNA and maintains genome stability during DNA replications stress [86]. Deletion of the Rfa1-binding domain in Bur1 renders cells sensitive to hydroxyurea and methanesulfonate [86].

## Cdk12 and Cdk13 in disease

Considering that Cdk12 regulates the expression of several cancer-related genes, such as *BRCA1 *[5,7], it comes as no surprise that the dysregulation of Cdk12 has been identified in several cancers. A comprehensive genomic approach identified *Cdk12 *to be one of the most frequently somatically mutated genes in high-grade serous ovarian cancer, the most fatal form of the disease [88]. Next to the nonsense and indel mutations that lead to the loss of protein function, several point mutations in the kinase domain have also been identified [88]. This finding points to the critical importance of the kinase activity of Cdk12 for the development/progression of this disease. Since about half of the ovarian cancer samples were defective in homologous recombination (HR) [88], we can speculate that the aberrant CTD kinase activity of Cdk12 results in the down-regulation of several HR regulators [5,7], and defective HR can lead to the development of the disease [7].

Several pieces of evidence also point to an important role for Cdk12 in the development of breast cancer. Notably, Cdk12 is located on chromosome 17, within the 17q21 locus that contains several candidate genes for breast cancer susceptibility [89,90], and it is co-amplified with the tyrosine kinase receptor ERBB2, a protein amplified and overexpressed in about 20% of breast tumors [91,92]. Gene fusion between *Cdk12 *and *ERBB2 *was also detected in gastric cancer [93]. Cdk12 is also implicated in the modification of tamoxifen sensitivity in estrogen-positive breast cancer via the modulation of the mitogen-activated protein kinase pathway [94]. Interestingly, decreased expression of BRCA1 was linked to the occurrence of sporadic breast cancer and is correlated with a poor prognosis for patients [95,96]; however, the mechanism of this aberrant expression is poorly understood.

Currently, less evidence exists for the clinical significance of Cdk13. Increased levels of Cdk13 were found in patients with refractory anemia with ringed sideroblasts, associated with marked thrombocytosis, a disease caused by ineffective hematopoiesis [97]. Another report demonstrates that Cdk13 is necessary for megakaryocyte development [98]. It has been suggested that Cdk13 affects the splicing of HIV and might act as its restriction factor [51] and that CycK inhibits HIV expression by interfering with CycT1/Cdk9 complex formation in a Nef-dependent manner [99].

Evidence is accumulating that the aberrant phosphorylation of CTD correlates with the onset and progression of many diseases (for example, cardiac hypertrophy [100], leukemia [101-103], and HIV [20,104,105]. Thus, the identification of Cdk12, along with Cdk9, as the major Ser2 kinases, makes these attractive candidate targets for the development of small chemical inhibitors as therapeutic agents. At present, approximately 30 compounds are known to inhibit Ser2 phosphorylation in the CTD [106]. Among them, DRB and flavopiridol are mostly used in research studies to inhibit Cdk9. It will be of great interest to validate the ability of these compounds to inhibit the Ser2 kinase activity of Cdk12 and to compare their effects with the inhibition of Cdk9. So far, it has been suggested that flavopiridol, the most specific inhibitor of Cdk9 [64,107], does not inhibit Cdk12 and Cdk13 in concentrations sufficient for the inhibition of Cdk9 [63].

## CycK in development

CycK complexes play a crucial role in embryo development, as genetic inactivation of *CycK *in mice leads to a lethal phenotype at the stage of the morula-blastocyst transition [5]. However, currently we can only speculate about the specific function of the CycK complexes in embryo development. It is possible that CycK complexes, through regulating phosphorylation of the CTD of RNAPII, direct the expression of genes important for the transition of individual developmental stages. Although, no aberrant expression of genes known to be involved directly in development was detected when CycK or Cdk12 were depleted in human cell lines [5], more physiologically relevant experiments in CycK knock-out mouse embryonic stem cells could better address this question. Genetic inactivation of other cyclin/Cdk complexes involved in CTD phosphorylation results in an embryonic lethal phenotype. For example, inactivation of *CycT2 *leads to the death of embryos at 4-cell stage and depletion of CycT2 in mouse embryonic stem cells affects the expression of Lefty1 and Lefty2, important regulators of early development [108]. Cdk8 was shown to be essential for preimplantation mouse development, perhaps by affecting the transcriptional repression of genes critical for an early cell fate determination [109]. Dysregulated expression of genes responsible for DDR and the regulation of basic processes in the cell could be another reason for the embryonic lethal phenotype of the CycK knock out mice [5]. In support of this hypothesis, knock out of some members of the DDR pathways, such as *ATR *[110,111] and *BRCA1 *[112], also lead to early embryonic lethality in mice. The monitoring of CycK expression in embryos by the activity of the *beta-galactosidase *gene under the control of an endogenous *CycK *promoter revealed that CycK is globally expressed in embryos at different embryonic stages [5]. This observation correlates with the proposed function of CycK in early embryo development. Interestingly, the most distinct signal was observed in the formation of neural tube and brain structures at embryonic day 8.5 [5], suggesting an important role for CycK in the process of neurogenesis. In agreement with this observation, CycK was identified as one of the factors necessary for the development of nervous system in *Drosophila *[113]. A study performed in *Xenopus laevis *showed that recruitment of CycK and CycT2 has different effects on the endoderm-inducing activity of the homeodomain protein, Mix.3 [114].

## Perspective

Although research of CycK, Cdk12, and Cdk13 is at an early stage, recent studies have already uncovered several pieces of evidence of these proteins' significant medical relevance. In the next few years, we should learn more about these proteins' roles in regulation of transcription, posttranscriptional mRNA processing, and other CTD RNAPII-regulated cellular functions. These studies should reveal more about the function of these proteins in cellular processes, human disease, and embryonic development.

## Competing interests

The authors declare that they have no competing interests.

## Authors' contributions

Both J.K. and D.B. contributed to writing this article. Both authors read and approved the final manuscript.
